# The impact of collateral therapeutics on stroke hemodynamics in normotensive and hypertensive rats: a step toward translation

**DOI:** 10.3389/fneur.2024.1373445

**Published:** 2024-03-21

**Authors:** Marilyn J. Cipolla, Ryan D. Hunt, David S. Liebeskind, Sarah M. Tremble

**Affiliations:** ^1^Department of Neurological Sciences, Larner College of Medicine, University of Vermont, Burlington, VT, United States; ^2^Department of Obstetrics, Gynecology and Reproductive Sciences, Larner College of Medicine, University of Vermont, Burlington, VT, United States; ^3^Department of Pharmacology, Larner College of Medicine, University of Vermont, Burlington, VT, United States; ^4^Department of Electrical and Biomedical Engineering, College of Engineering and Mathematical Sciences, University of Vermont, Burlington, VT, United States; ^5^Department of Neurology, University of California Los Angeles, Los Angeles, CA, United States

**Keywords:** ischemic stroke, collateral circulation, hypertension, treatment, autoregulation

## Abstract

**Introduction:**

Stroke interventions that increase collateral flow have the potential to salvage penumbral tissue and increase the number of patients eligible for reperfusion therapy. We compared the efficacy of two different collateral therapeutics during transient middle cerebral artery occlusion (tMCAO) in normotensive and hypertensive rats.

**Methods:**

The change in collateral and core perfusion was measured using dual laser Doppler in response to either a pressor agent (phenylephrine, 10 mg/kg iv or vehicle) or a collateral vasodilator (TM5441, 5 mg/kg iv or vehicle) given 30 min into tMCAO in male Wistar and spontaneously hypertensive rats (SHRs).

**Results:**

Pressor therapy increased collateral flow in the Wistar rats but was ineffective in the SHRs. The increase in collateral flow in the Wistar rats was associated with impaired cerebral blood flow autoregulation (CBFAR) that was intact in the SHRs. TM5441 caused a decrease in collateral perfusion in the Wistar rats and a modest increase in the SHRs. The pressor therapy reduced early infarction in both groups but increased edema in the SHRs, whereas TM5441 did not have any beneficial effects in either group.

**Conclusions:**

Thus, the pressor therapy was superior to a collateral vasodilator in increasing collateral flow and improving outcomes in the Wistar rats, likely due to pial collaterals that were pressure passive; the lack of CBF response in the SHRs to pressor therapy was likely due to intact CBFAR that limited perfusion. While TM5441 modestly increased CBF in the SHRs but not in the Wistar rats, it did not have a beneficial effect on stroke outcomes. These results suggest that collateral therapies may need to be selected for certain comorbidities.

## 1 Introduction

The secondary pial collateral system in the brain is one of the most important means of limiting ischemic injury during a large vessel occlusion (LVO). The pial or leptomeningeal anastomoses (LMAs) redirect blood from the unoccluded to the occluded vascular territory, thereby increasing cerebral blood flow (CBF) to the penumbra, potentially salvaging brain tissue otherwise destined for infarction (1, 2). The pial collateral network is also predictive of outcome, as patients with good collateral on imaging have a better outcome than those with poor collateral (3, 4). In addition, while mechanical thrombectomy has become the standard of care for LVO, there is still a need to expand the inclusion of patients at extremes of time and infarct size that do not meet the imaging requirements (5–7). Importantly, even in prospective clinical trials of mechanical thrombectomy in the late time window, adequate collateral flow was still an important consideration (8).

The ability to increase collateral flow during an occlusion (“collateral therapeutics”) could prevent the inevitable progression of infarction and potentially expand the number of patients eligible for mechanical thrombectomy, especially in patients with large infarct cores who present late in the time window. In addition, collateral-enhancing procedures may provide an important route to administer cerebroprotective agents that can improve outcomes from LVO. Numerous collateral therapeutics have been in clinical trials with limited success. For example, sphenopalatine ganglion stimulation (SPG) showed benefit in patients even when performed at 18 h after symptom onset but was location-specific (9). Pressor therapy, or therapeutic-induced hypertension, has been used in the neurocritical care setting, including for the treatment of acute ischemic stroke. In a clinical trial of pressor therapy for the treatment of acute ischemic stroke, there was an early neurological improvement in a subset of patients (58% showed benefit) (10). Partial aortic occlusion has also been used as a means to augment CBF during occlusion. In a clinical trial (SENTIS), aortic occlusion showed benefit only in a subset of patients with moderate stroke severity when treated within 6 h of symptom onset (11–13). Other therapeutic means to increase collateral flow either showed no benefit or have only been successfully tried in animal studies (14–16).

That collateral-enhancing therapies have shown benefits in subsets of patients highlights our limited understanding of collateral flow and its impact on cerebral hemodynamics. Transgenic mouse studies showed that the number and size of LMAs critically determine the size of infarction (17), which from a hemodynamic standpoint makes sense given that lumen diameter is the most powerful determinant of blood flow (18). However, we previously showed that LMAs are vasoactive and respond to increases in pressure with myogenic vasoconstriction that was pronounced in a model of chronic hypertension (19, 20). LMAs are high-resistance distal connections between large artery territories. The efficacy of collateral therapeutics likely depends on the response of these vessels and their impact on stroke hemodynamics, as these vessels are the therapeutic target. For example, pressor therapy as a collateral therapeutic involves increasing systemic blood pressure with a pressor agent such as phenylephrine that hemodynamically enhances CBF through increased cerebral perfusion pressure. However, CBF will only increase in response to increased systemic blood pressure if the collateral circulation is pressure passive, i.e., CBF autoregulation (CBFAR) is impaired. This may not be the case in chronic hypertension, in which LMAs are vasoconstricted (20, 21). Conversely, collateral therapies that promote the vasodilation of LMAs would be most effective in patients in whom pial collaterals are vasoconstricted, such as in chronic hypertension. Thus, the vasoconstricted state of LMAs and their vasoactive response to collateral therapeutics in various comorbid conditions may be one of the reasons collateral therapies show benefit only to subsets of patients. Another factor that may significantly impact the efficacy of collateral therapy is the state of CBFAR in the penumbra, which could either enhance or counteract CBF augmentation, especially with an increase in cerebral perfusion pressure.

We aimed to understand the impact of two different collateral therapies on CBF hemodynamics in the core middle cerebral artery (MCA) and anterior cerebral artery (ACA) collateral vascular territories in both normotensive Wistar rats and spontaneously hypertensive rats (SHRs). The SHR is a model of chronic hypertension and presents with a large ischemic core with little salvageable tissue during stroke (22, 23). We compared pressor therapy to a vasodilator—infusion of TM5441, a small molecule inhibitor of plasminogen activator inhibitor-1 (PAI-1) that enhances the collateral flow through increased nitric oxide (NO) (19, 24). We hypothesized that the pressor therapy would be more effective in normotensive animals in which pial collaterals are relatively pressure passive (19), but that TM5441, a vasodilator, would be less effective. In addition, we hypothesized that the pressor therapy would be detrimental in the SHRs by limiting collateral perfusion due to effective CBF autoregulation, but that TM5441 would increase collateral flow due to constricted LMAs. All treatments were given 30 min into occlusion using the filament model of LVO. Finally, CBF autoregulation effectiveness was determined in the core MCA and ACA collateral territories to better understand stroke hemodynamics in normotensive and hypertensive rats.

## 2 Materials and methods

### 2.1 Animal model of stroke

All animal procedures were approved by the Institutional Animal Care and Use Committee at the University of Vermont, an Association for Assessment and Accreditation of Laboratory Animal Care International (AAALAC) accredited facility. Procedures were conducted in accordance with the National Institutes of Health Guide for the Care and Use of Laboratory Animals. Male Wistar rats and SHRs aged 12–32 weeks were used for this study and purchased from Charles River Canada. Male animals were used because we previously showed there was no sex difference in LMA structure or function (25). The animals were housed in pairs with enrichment, maintained on a 12-h light/dark cycle, and allowed access to food and water *ad libitum*. The rats were randomly assigned to the treatment group, and the experiments were conducted in a randomized fashion. ARRIVE 2.0 guidelines were followed for the reporting of results.

A proximal filament occlusion model of transient MCA occlusion (tMCAO) was used to model LVO, as previously described (19). The animals were anesthetized with isoflurane 2% in oxygen initially for instrumentation, after which it was decreased to ≤1.5% in oxygen for the remainder of the experiment. The animals were intubated and ventilated to maintain blood gases within physiological ranges. The animals in the pressor therapy group had 2 h of occlusion, and the animals in the TM5441 treatment group had 3 h of occlusion. All animals had 1 h of reperfusion. The difference in ischemic duration was because our previous study showed TM5441 increased collateral flow and improved outcome in SHRs after 2 h of occlusion (15) and therefore we increased the ischemic duration in the current study to determine if TM5441 would confer benefit after a longer period of occlusion to mimic a late time window.

### 2.2 Treatments and measurement of CBF

For the pressor therapy, Wistar rats (*n* = 17) and SHRs (*n* = 12) were given PE (Sigma-Aldrich, St. Louis, MO, 10 mg/mL in saline) or saline (vehicle) infusion intravenously after 30 min of filament occlusion to increase baseline blood pressure by ~30%. The PE or saline infusion was for 10 min, after which the infusion was stopped. For the TM5441 treatment, separate sets of Wistar (*n* = 17) and SHRs (*n* = 17) were treated intravenously with TM5441 (Tocris, Bristol, UK, 5 mg/kg in DMSO and 20% Captisol) or vehicle (20% Captisol in DMSO, MedChemExpress, Monmouth Jct, NJ) after 30 min of occlusion for 10 min, after which infusion was stopped. A separate set of Wistar (*n* = 4) and SHRs (*n* = 4) were instrumented and treated with TM5441 as described above, except that a combination probe that measured oxygen and blood flow (OxyFlo/OxyLite, Oxford Optronix, United Kingdom) was used to measure cortical CBF in the penumbra and brain tissue oxygen (btO_2_). The combined oxygen/Doppler flow probe was placed 2.75 mm into the expected peri-infarct brain region through a burr hole in the lateral parietal bone and aligned with zero Bregma at a depth of 2 mm. Supplementary Figure S1 shows probe placement for the combined oxygen/Doppler flow probe.

Blood pressure was measured continuously and simultaneously with MCA and ACA collateral CBF before, during, and after treatment or vehicle by a femoral artery catheter and an inline pressure transducer (Living Systems Instrumentation, Burlington, VT). Changes in CBF were measured in two vascular territories simultaneously—the MCA core and MCA-ACA collateral territory—using two laser Doppler probes (Perimed Ardmore, PA) placed +4 mm lateral of midline and − 2 mm posterior of Bregma for MCA flow and + 3 mm lateral of midline and + 2 mm anterior of Bregma for collateral flow measurements. Supplementary Figure S1 shows the placement of both laser Doppler probes. The effectiveness of CBF autoregulation was determined in each group of Wistar and SHRs given the pressor therapy by analyzing the relationship between CBF and the change in blood pressure, which was sampled every minute and averaged. The stronger the correlation between blood pressure and CBF, the less effective the autoregulation.

### 2.3 Infarct and edema measurements

Measurements were made by investigators who were blinded to the experimental groups. At the completion of reperfusion, the animals were euthanized by rapid decapitation under isoflurane anesthesia (2% oxygen). The brains were removed and coronally sectioned into 2 mm slices for the measurement of infarction. Infarct volume was determined by 2,3,5-triphenyltetrazolium chloride (TTC) staining, identified as the white area of each section, and measured with ImageJ software (NIH, Bethesda, MD). Infarct volume was calculated after subtracting the contralateral area from the ipsilateral area in the section to correct for swelling, i.e., edema. The difference between contralateral and ipsilateral areas was used as a measure of edema.

### 2.4 Excluded animals

Four animals were removed due to hemorrhaging (two Wistar rats treated with pressor and two Wistar rats treated with vehicle in the TM5441 group). Five animals were removed for being statistical outliers in the CBF measurements (two Wistar rats treated with vehicles, two Wistar rats treated with TM5441, and one SHR treated with TM5441). All animals were included in the infarct and edema analyses, except for those that hemorrhaged.

### 2.5 Data calculations and statistical analysis

The change in CBF was determined by calculating the % change from baseline or just prior to treatment. Results are presented as mean ± SEM. Statistical analysis was conducted using GraphPad Prism 10.1.2 software (GraphPad Software Inc., La Jolla, CA). The normal distribution of data was determined using Kolmogorov–Smirnov or D’Agostino and Pearson normality tests. Differences between SHRs and Wistar rats or treated and vehicle groups were determined by either multiple t-tests or multiple Mann–Whitney tests. A two-way repeated measures analysis of variance (ANOVA) was used to determine differences in infarction and edema. Pearson’s *r* correlation was used to determine the correlation between changes in CBF and blood pressure. Differences were considered significant at a *p*-value of <0.05. The number of animals used in each experiment was determined by statistical power calculations based on our previous studies using similar methodologies (19, 26).

## 3 Results

### 3.1 Effect of pressor therapy

**Figure 1:**
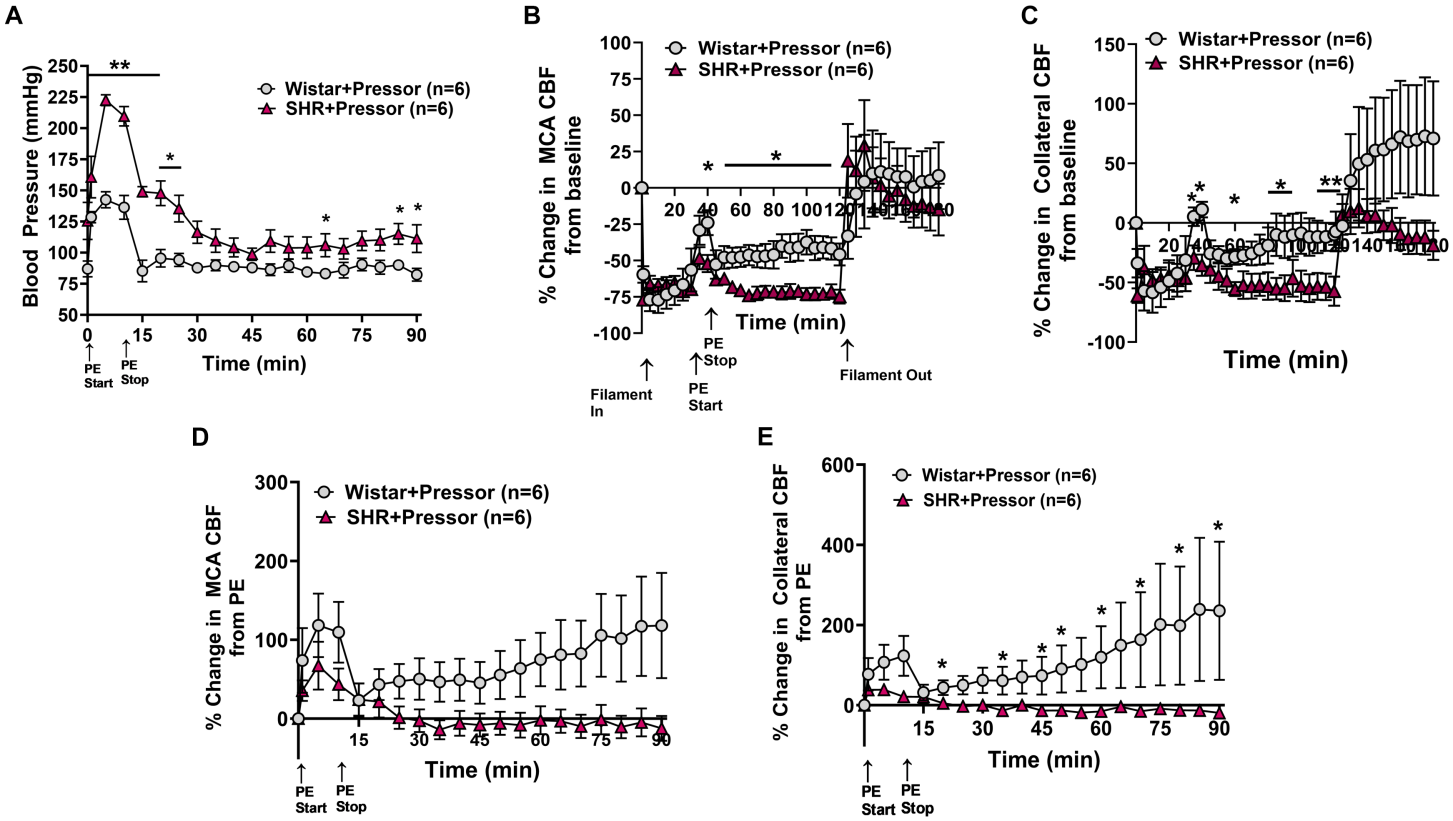
Effect of pressor therapy on blood pressure and changes in CBF in the Wistar rats and SHRs. **(A)** Blood pressure in response to PE infusion as a pressor therapy during 90 min of occlusion. PE infusion increased blood pressure in both groups, which was greater in the SHRs. **(B)** Percent change in MCA CBF from baseline in the Wistar rats vs. the SHRs over 2 h of ischemia and 1 h of reperfusion. There was an increase in MCA CBF with PE infusion in both groups, which was higher in the Wistar rats than the SHRs and remained elevated. **(C)** Percent change in collateral CBF from baseline in the Wistar rats vs. SHRs over 2 h of ischemia and 1 h of reperfusion. The Wistar rats had a significant increase in CBF with PE infusion that was not seen in the SHRs. Collateral perfusion remained elevated in Wistar rats but not in the SHRs. **(D)** Percent change in MCA CBF from PE infusion in Wistar rats vs. the SHRs. PE infusion increased MCA CBF in both groups, while blood pressure remained high. In the Wistar rats, MCA perfusion increased over time, whereas flow decreased in the SHRs. **(E)** Percent change in collateral CBF from PE infusion in the Wistar rats vs. the SHRs. PE infusion increased collateral flow in the Wistar rats but not the SHRs, which also increased over time. *p < 0.05 and **p < 0.01 vs. SHRs.

Figure 1A shows the change in blood pressure in the Wistar rats and SHRs at the start of PE infusion and for 90 min of ischemia. PE increased blood pressure in both groups of animals for the duration of the infusion which was greater in SHRs, demonstrating an increased sensitivity to pressor agents in this model (27). Figures 1B,C show the % change in MCA and ACA collateral perfusion, respectively, calculated from baseline CBF in both groups of animals. PE infusion increased MCA and collateral flow in both Wistar and SHRs; however, the increase in flow was greater in the Wistar rats despite a smaller increase in blood pressure, suggesting less effective CBF autoregulation. In addition, the increase in MCA and collateral flow was transient in both groups to a varying degree—CBF returned to baseline in the SHRs whereas it remained elevated in the Wistar rats, suggesting a lasting effect of pressor therapy on CBF in that group. Furthermore, while there did not appear to be a difference between groups in MCA flow during reperfusion, collateral CBF was above baseline in the Wistar rats during reperfusion and below baseline in the SHRs, which continued to decrease. Figures 1D,E shows the change in MCA and collateral flow calculated just before PE infusion during occlusion in both groups during 90 min of ischemia to isolate the response to PE while the filament was in place. The effect of pressor therapy on MCA and collateral CBF was distinctly different between groups. Supplementary Figure S2 shows representative tracings of blood pressure, and changes in MCA and collateral flow. MCA flow increased after blood pressure returned to baseline in the Wistar rats but decreased in SHRs. Collateral CBF increased to a greater extent in the Wistar rats in response to PE that returned to baseline when PE was stopped and pressure returned to normal; however, over time, collateral flow increased in the Wistar rats and declined in the SHRs.

**Figure 2:**
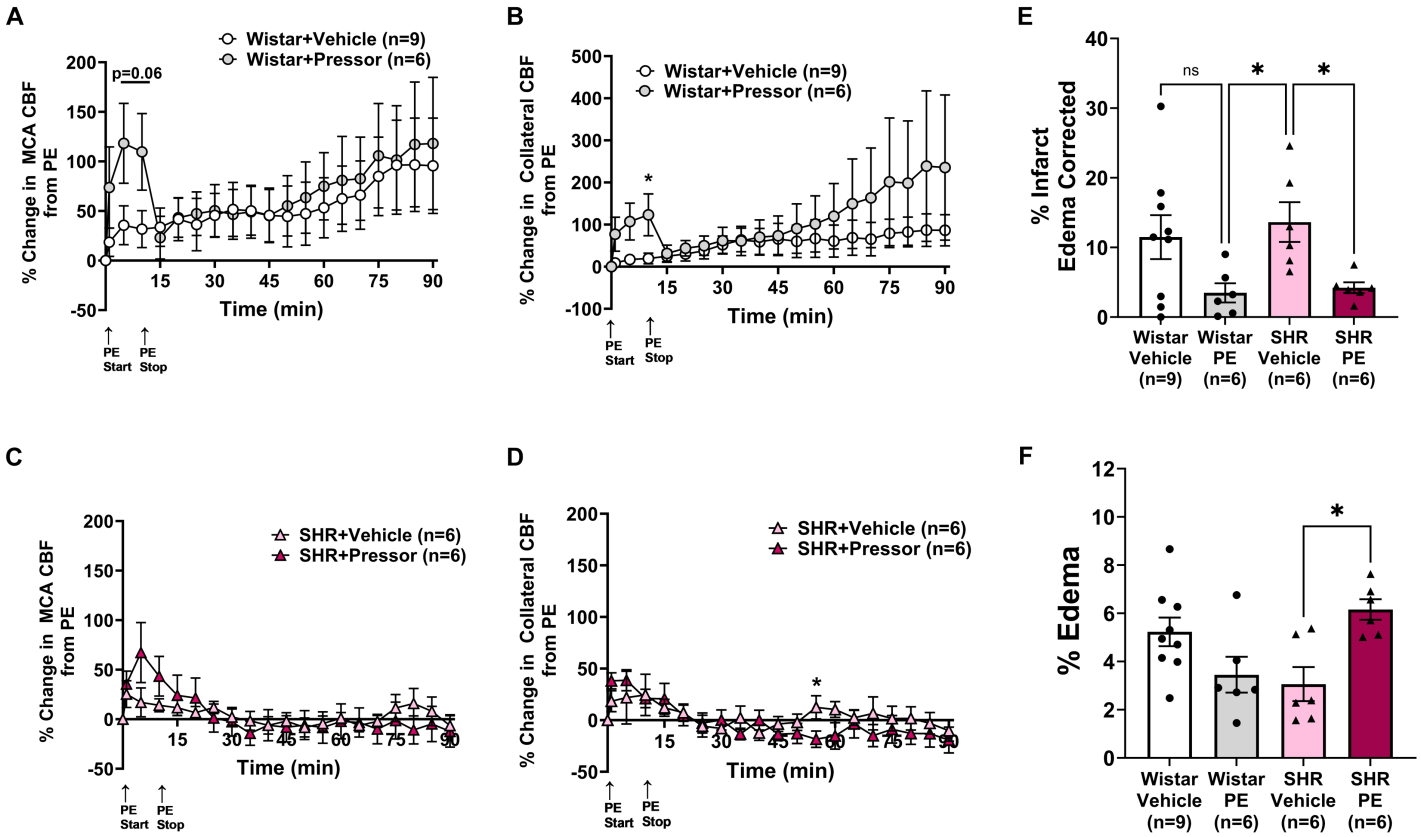
Effect of pressor therapy on changes in CBF, infarction, and edema. Percent change in **(A)** MCA CBF or **(B)** collateral CBF from PE or vehicle infusion in Wistar rats. Percent change in **(C)** MCA CBF or **(D)** collateral CBF from PE or vehicle infusion in the SHRs. **(E)** % infarction and **(F)** % edema in all groups of treated animals. *p < 0.05 vs. vehicle.

To determine the effect of pressor therapy on CBF and stroke outcome, we compared the infusion of PE to a vehicle (Figure 2). Compared to PE infusion, the vehicle treatment had less effect on the change in MCA and collateral flow in the Wistar rats (Figures 2A,B). Interestingly, the increase in MCA flow over time during ischemia did not appear to be related to the increase in blood pressure, as flow increased similarly in the vehicle-treated rats. Collateral flow in the Wistar rats increased in response to increased blood pressure with PE compared to the vehicle and variably increased over time. In the SHRs, PE infusion to raise blood pressure transiently increased MCA flow to a much lesser degree than the Wistar rats, but not collateral flow (Figures 2C,D). In contrast to the Wistar rats, neither MCA nor collateral flow increased over time. Figures 2E,F shows % infarction and edema after 2 h of occlusion and 1 h of reperfusion in all groups. In the Wistar rats, PE treatment non-significantly decreased infarction with little effect on edema. In the SHRs, infarction was decreased with PE compared to the vehicle, but edema was increased. Representative TTC images of coronal sections are shown in Supplementary Figure S3.

### 3.2 Autoregulation of CBF

**Figure 3:**
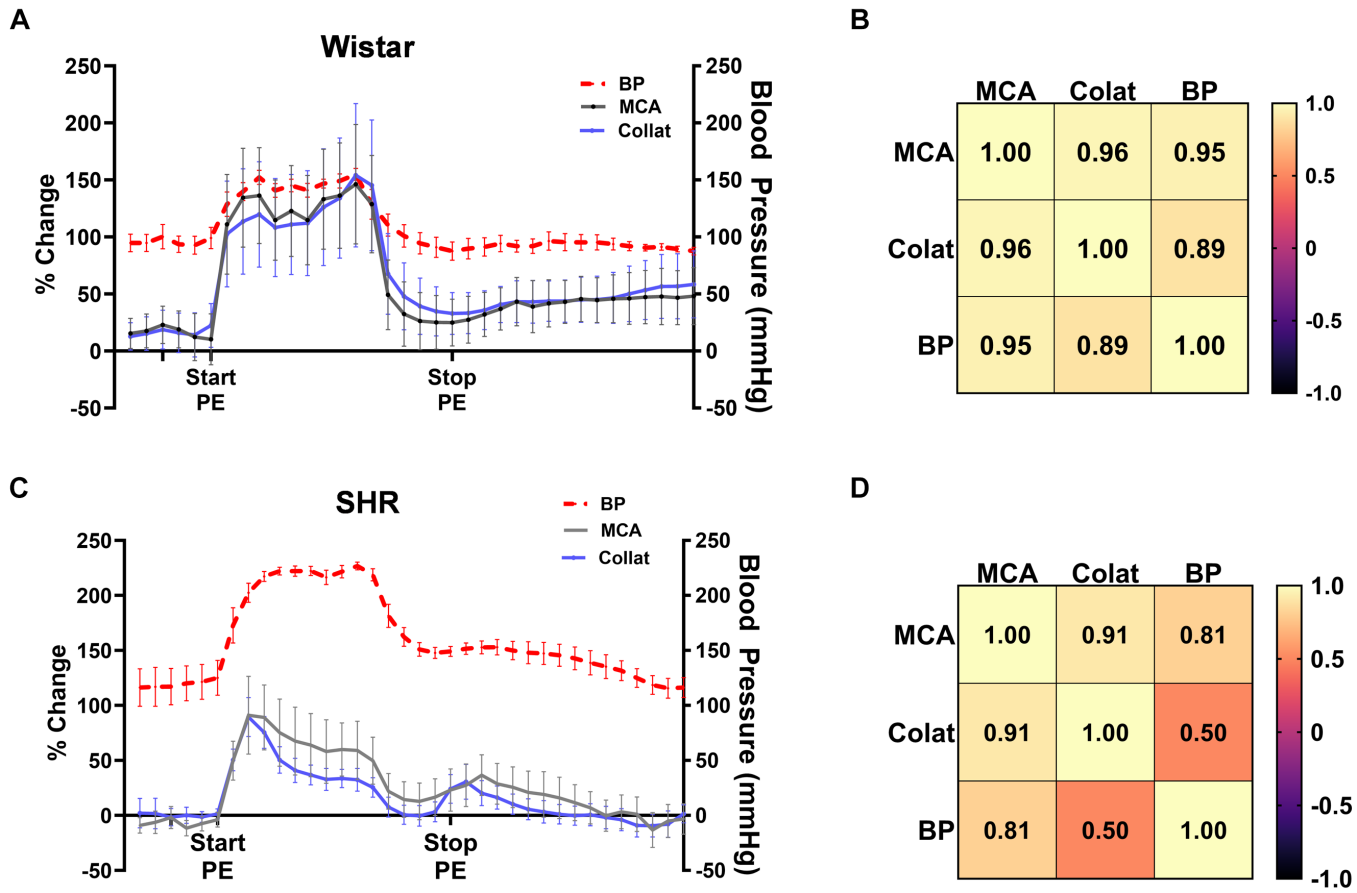
CBF autoregulation during ischemia in the Wistar rats and SHRs. **(A)** Percent change in MCA and collateral flow together with corresponding blood pressure in the Wistar rats during ischemia. **(B)** Pearson’s correlation matrix shows the correlation between MCA and collateral CBF with pressure as a measure of CBF autoregulation effectiveness. **(C)** Percent change in MCA and collateral flow together with corresponding blood pressure in the SHRs during ischemia. **(D)** Pearson’s correlation matrix shows the correlation between MCA and collateral CBF with pressure as a measure of CBF autoregulation effectiveness during ischemia.

Pressor therapy relies on CBF autoregulation to be impaired in the affected territory during stroke, most notably the collateral circulation as the penumbra is the target of treatment. To determine the effectiveness of CBF autoregulation in the MCA and collateral circulations, we correlated MCA and collateral flow with blood pressure in Wistar rats and SHRs (Figure 3). Figure 3A shows the change in MCA and collateral CBF along with the corresponding blood pressure in the Wistar rats. Notice that MCA and collateral flow both increased with increased blood pressure and remained elevated during PE infusion. CBF in both territories returned almost to baseline when blood pressure returned to pre-PE levels. Pearson’s correlation matrix shown in Figure 3B shows a good correlation between the change in both MCA (*r* = 0.95) and collateral flow (*r* = 0.89) with blood pressure, demonstrating impaired CBF autoregulation in the Wistar rats during occlusion, i.e., CBF was mostly pressure passive. Figure 3C shows the same relationship between CBF and blood pressure in SHR. Notice that CBF transiently increased with increased blood pressure. Pearson’s correlation matrix shown in Figure 3D shows a modest correlation between the change in MCA flow and blood pressure in the SHRs (*r* = 0.81) and less correlation between the change in collateral blood flow and blood pressure (*r* = 0.50), suggesting that autoregulation was mostly intact in the SHRs in the collateral circulation during ischemia.

### 3.3 TM5441 treatment

**Figure 4:**
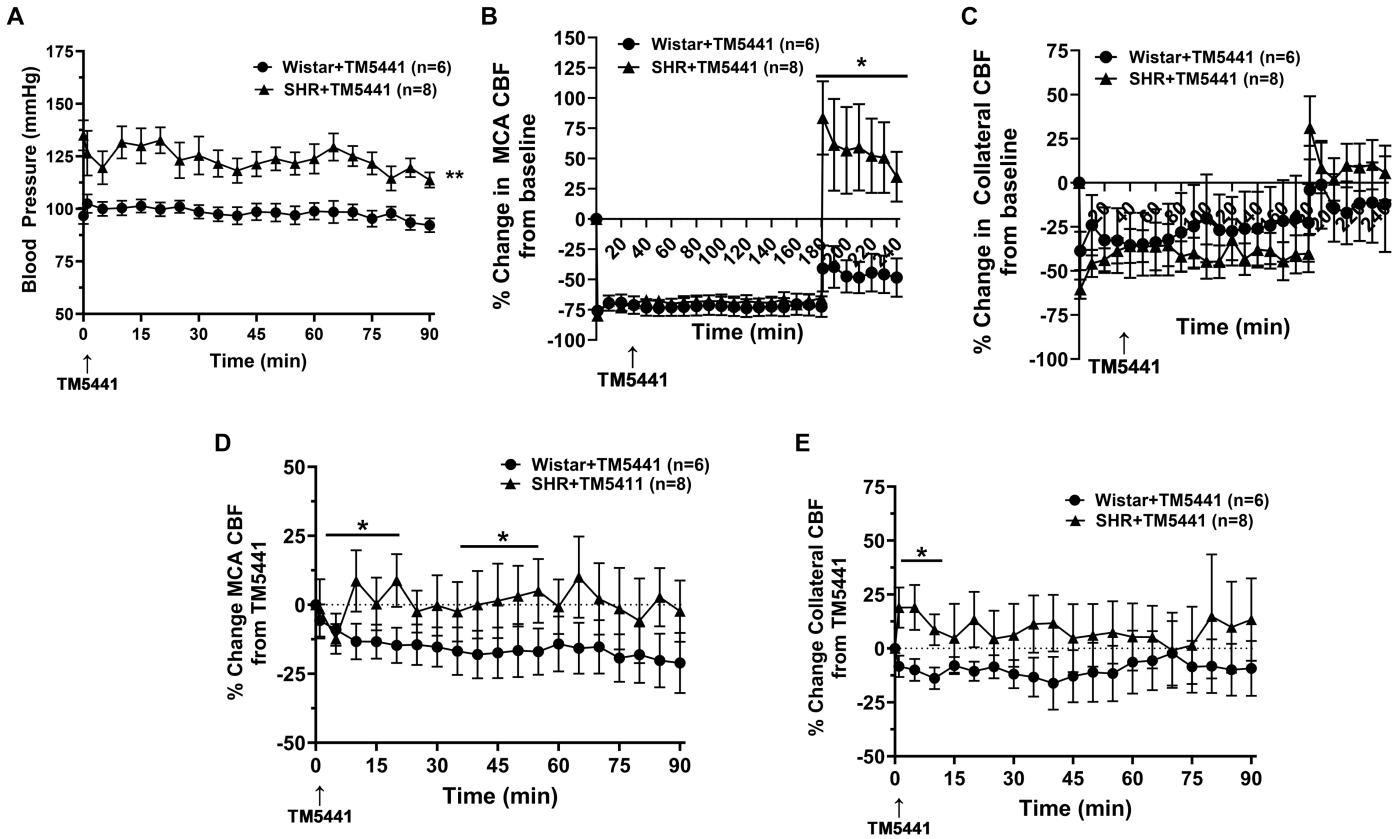
Effect of TM5441 treatment on blood pressure and changes in CBF in the Wistar rats and SHRs. **(A)** Blood pressure did not change in either group with the TM5441 infusion. **(B)** Percent change in MCA CBF from baseline in the Wistar rats vs. the SHRs over 3 h of ischemia and 1 h of reperfusion. There was little change in MCA CBF with TM5441 infusion in either group during ischemia. However, TM5441 increased MCA flow during reperfusion compared to the Wistar rats. **(C)** Percent change in collateral CBF from baseline in the Wistar rats vs. SHRs over 3 h of ischemia and 1 h of reperfusion. TM5441 decreased MCA flow in the Wistar rats but not in the SHRs. **(D)** Percent change in MCA CBF from TM5441 infusion in the Wistar rats vs. SHRs. **(E)** Percent change in collateral CBF from TM5441 infusion in the Wistar rats vs. the SHRs. TM5441 infusion increased collateral flow in the SHRs during the first 10 min of infusion, but not the Wistar rats, which had a flow that decreased compared to prior to infusion. *p < 0.05 and **p < 0.01 vs. SHR.

Infusion of the PAI-1 inhibitor TM5441 was used as a collateral therapeutic that causes vasodilation of pial collaterals (19). Figure 4 shows the effect of TM5441 given 30 min into ischemia in the Wistar rats and SHRs. TM5441 did not appear to affect the blood pressure, which was significantly higher in the SHRs than in the Wistar rats (Figure 4A). When calculated from baseline, TM5441 did not have much effect on MCA or collateral CBF in either group during ischemia (Figures 4B,C). However, TM5441 increased reperfusion in the SHRs compared to the Wistar rats, which was more pronounced in the MCA territory. Supplementary Figure S4 shows representative tracings of blood pressure and changes in MCA and collateral flow. When the change in MCA CBF was calculated during occlusion from just prior to TM5441 infusion, treatment with TM5441 decreased CBF in the Wistar rats, an effect not seen in the SHRs that was higher than the Wistar rats (Figure 4D). TM5441 also decreased collateral flow in the Wistar rats but increased collateral flow in the SHRs within the first 10 min and returned to levels prior to infusion (Figure 4E).

**Figure 5:**
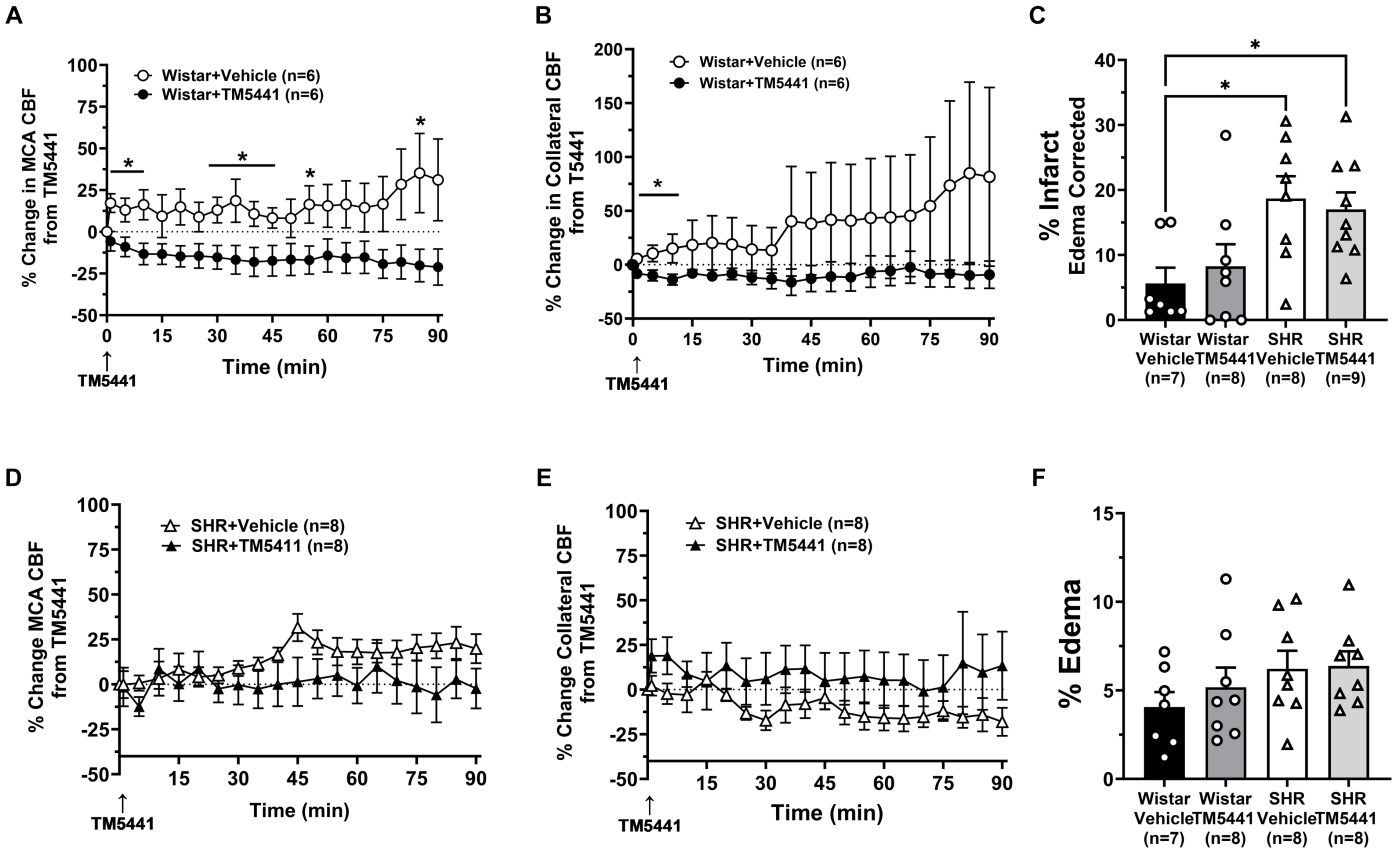
Effect of TM5441 treatment on changes in CBF, infarction, and edema. Percent change in **(A)** MCA CBF and **(B)** collateral CBF from TM5441 treatment or vehicle infusion in the Wistar rats. Percent change in **(D)** MCA CBF and **(E)** collateral CBF from TM5441 or vehicle infusion in the SHRs. **(C)** % infarction and **(F)** % edema in all groups of treated animals. *p < 0.05 vs. vehicle.

To investigate the effect of TM5441 on CBF and stroke outcome, we compared the treatment to a vehicle. Figure 5 shows the change in CBF in the Wistar rats and SHRs during occlusion just prior to the TM5441 infusion compared to the vehicle. In the Wistar rats, TM5441 decreased MCA and collateral CBF. In contrast, vehicle treatment in the Wistar rats caused little change in MCA and a variable increase in collateral flow (Figures 5A,B). In the SHRs, TM5441 treatment had no effect on MCA CBF compared to the vehicle. However, in vehicle-treated SHRs, collateral flow decreased over time, an effect that was not seen with TM5441 treatment (Figures 5D,E). Figures 5C,F shows percent infarction and edema in both groups treated with TM5441 or vehicle. Treatment with TM5441 did not have an effect on either group compared to the vehicle. Infarction and edema were significantly greater in the SHRs compared to the Wistar rats, regardless of treatment. Representative TTC images of coronal sections are shown in Supplementary Figure S3.

**Figure 6:**
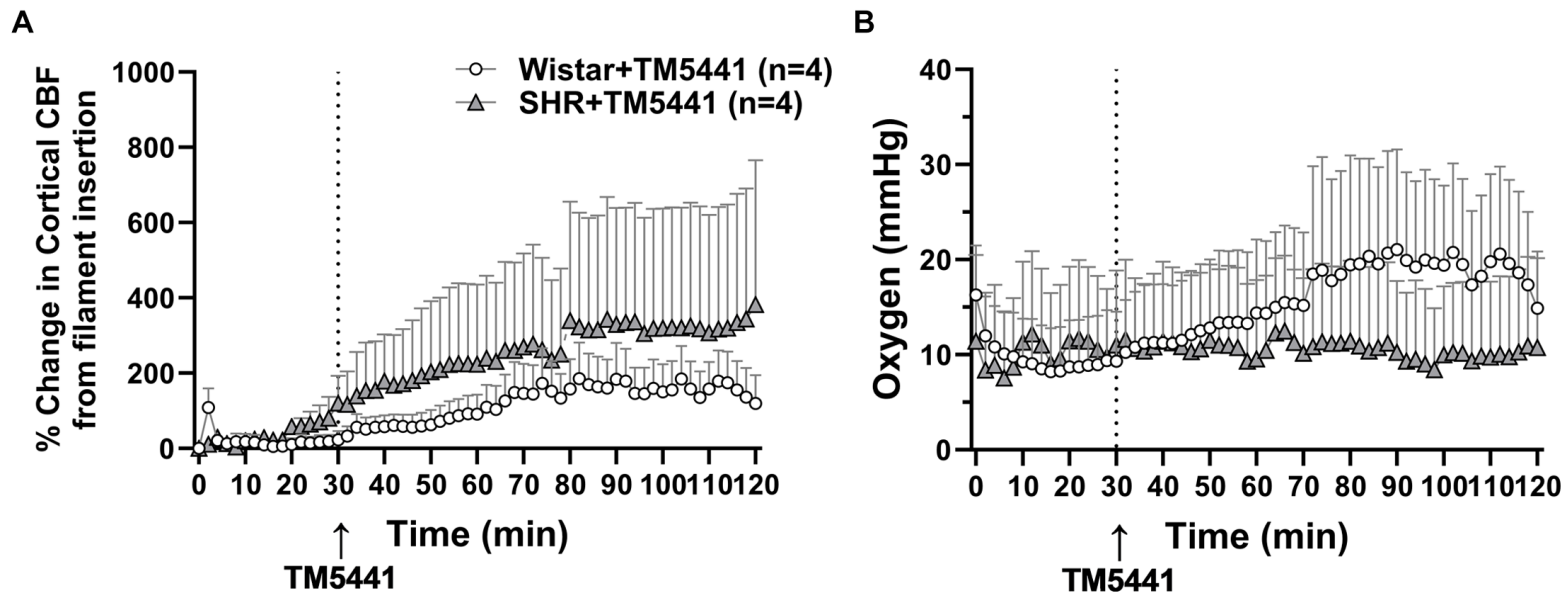
Effect of TM5441 treatment on changes in cortical CBF and oxygenation in the penumbra of the Wistar rats and SHRs. **(A)** Percent change in cortical CBF and **(B)** brain tissue oxygenation.

TM5441 treatment appeared to have a greater effect on CBF in the SHRs, yet there was no apparent benefit of treatment. Thus, to better understand the effect of TM5441 on brain tissue CBF (as opposed to pial CBF) in the peri-infarct region and btO_2_, we used a combination probe that measured cortical CBF and oxygenation spatially and temporally linked within the expected penumbra. Figure 6A shows the change in cortical CBF calculated from filament insertion, and Figure 6B shows btO_2_ in the same brain region. TM5441 appeared to increase cortical CBF in both Wistar rats and SHRs, which was greater in the SHRs, similar to the LMA flow shown in Figure 5. Interestingly, TM5441 increased oxygenation within the penumbra to a greater extent in the Wistar rats. In fact, penumbra oxygenation in the SHRs was low and remained unchanged despite the increase in cortical CBF.

## 4 Discussion

Stroke interventions that target increasing collateral perfusion are a promising means to salvage penumbral tissue and increase the number of patients eligible for reperfusion therapies. An important aspect of successful treatments that target collateral perfusion is our understanding of the hemodynamics of the collateral circulation, especially during LVO. The collateral circulation is unique in that it operates under low (bidirectional) flow and low shear stress normally and is subjected to substantial retrograde increases in flow and shear stress during a LVO (2, 28, 29). In the current study, we compared two different collateral therapies given during occlusion of the MCA in two different animal models, the Wistar and SHRs. These models were chosen because they have LMAs with different vasoactivity and collateral perfusion. For example, LMAs from the Wistar rats have little myogenic tone and respond modestly to increases in pressure with vasoconstriction (19). In contrast, LMAs from the SHRs, a model of chronic hypertension, have a substantial myogenic tone and respond to pressure with robust vasoconstriction (19). The response to MCA occlusion is also different between these models in that SHRs have little salvageable tissue and large infarcts compared to the Wistar rats. We therefore compared two different collateral therapies to determine the effectiveness of these two different models. The collateral therapies we chose to investigate included a pressor therapy that relies on a hemodynamic increase in cerebral perfusion pressure from increased systemic blood pressure. PE was used as a pressor agent because it does not readily cross the BBB and there are few adrenergic receptors on pial arteries in the brain and none on LMAs (19, 30). The pressor therapy was compared to a collateral vasodilator, TM5441, that increases collateral perfusion by NO release from LMAs (19).

There are several explanations for the apparent differences in CBFAR effectiveness in the different animal models. In the Wistar rats, it is likely that perfusion pressure in both MCA and collateral vascular territories was below the myogenic pressure range, and therefore, both circulations were mostly pressure passive. In the SHRs, it is possible that perfusion pressure in the MCA territory was below the myogenic pressure range and not in collateral vessels that responded myogenically to pressure with vasoconstriction and intact CBFAR. The implication of this result is that pressor therapy as a collateral therapy may be ineffective in SHRs due to intact CBFAR in the penumbra. The effectiveness of CBFAR during and after stroke is of considerable clinical interest and has been measured in numerous previous studies. Studies measuring dynamic CBFAR in patients with large MCA strokes have shown impaired CBFAR on the ipsilateral compared to the contralateral hemisphere. This impairment deteriorated over several days and was associated with delayed recovery (31–37). Impaired dynamic CBFAR is also associated with unfavorable outcomes, including a greater risk of developing cerebral edema and hemorrhagic transformation (38–41). Conversely, preserved dynamic CBFAR in the acute phase of stroke was an independent predictor of functional independence at 3 months and was associated with smaller infarct volumes (2, 28, 42). However, studies of static CBFAR, in which blood pressure is lowered over minutes to hours, have found mostly intact autoregulation in the ischemic core and penumbra (43, 44). Intact static but impaired dynamic CBFAR, in which CBF is measured in response to sudden rapid changes in blood pressure, has been noted previously (45) but the implications are unclear. However, understanding CBFAR in the acute phase of stroke could have a significant impact on the acute management and effectiveness of collateral therapeutics, especially those that target CBF augmentation by increasing system blood pressure, such as pressor therapy.

Interestingly, despite the lack of increase in collateral perfusion in the SHRs with PE, there was a beneficial effect on early infarction (Figure 2). It is possible that collaterals from the posterior cerebral arteries (PCAs) were recruited but not measured. In a previous study by Shin et al. that used the pressor therapy in C57b6 mice, an increase in both ACA-MCA and PCA-MCA collateral flow was found (46). In addition, the increase in collateral flow with pressor therapy was associated with an increase in oxygenation and cerebral metabolic rate of oxygen (CMRO), demonstrating the powerful effect of pressor therapy. However, that study, as well as other studies that have shown the beneficial effects of pressor therapy, were performed on animals without comorbidities. The difference in CBF responsiveness to PE between the Wistar and SHRs underscores the need to understand collateral perfusion during stroke in comorbid and aging models that more closely mimic stroke demographics. Importantly, despite a decrease in infarction, edema formation was significantly greater in the SHRs with pressor therapy, a known consequence of increased blood pressure in stroke patients.

The effect of a collateral vasodilator was also investigated in the current study in the Wistar and SHRs. We used TM5441, a small molecule inhibitor of PAI-1 that we previously showed increased collateral flow in the SHRs through NO release (19, 24). Wistar rats were not studied previously. In this study, we hypothesized that TM5441 would be less effective at increasing collateral flow in the Wistar rats since the collateral circulation was already dilated, i.e., pressure passive. Unlike PE, the infusion of TM5441 did not change blood pressure in either group of animals. Surprisingly, TM5441 appeared detrimental in the Wistar rats and decreased CBF compared to the SHRs and vehicle-treated group (Figures 4, 5). The mechanism for the decreased perfusion with TM5441 is not clear but could possibly be related to vasodilation in other vascular beds that shunted blood away from the brain. However, if this were the case, a decrease in blood pressure would be expected, but that did not occur. TM5441 did increase collateral flow in the SHRs for the first 10 min after infusion, a result similar to our previous study (19), but did not affect MCA flow. Compared to the Wistar rats, CBF in the SHRs was greater with TM5441 treatment, including during reperfusion, suggesting a vasodilatory effect only in the SHRs. Regardless, TM5441 did not have a beneficial effect on infarct in either group that was higher in the SHRs. Edema was also unchanged in all groups, unlike pressor therapy, which increased edema in the SHRs. Because TM5441 increased collateral flow in the SHRs but without apparent benefit to the outcome, we further investigated the change in cortical CBF and btO_2_ within the penumbra. Surprisingly, cortical CBF increased in the SHRs and to a lesser extent in the Wistar rats, but only the Wistar rats had an increase in oxygenation. It is possible that the SHRs had a higher CMRO that metabolically used oxygen faster than in the Wistar rats, leading to less available oxygen. Although this interpretation is speculative, CMRO may be another variable that needs to be considered for collateral therapeutics.

There are several limitations to this study. First, isoflurane was used as an anesthetic that is known to be a cerebral vasodilator. However, during MCAO, isoflurane was ≤1.5%, a level that others have shown has minimal effects on CBF and is similar to chloral hydrate (46). In addition, the SHRs had intact CBFAR in the collateral circulation, demonstrating that isoflurane did not cause substantial vasodilation. Second, we measured infarct and edema after only 3–4 h of ischemia and reperfusion and therefore do not know the effect of any treatment on final infarct volume. Long-term survival studies were not performed due to the high morbidity associated with arterial catheterization and dual laser Doppler probe placement in burr holes in the skull. Long-term studies are needed to determine the effectiveness of either collateral therapy. In addition, dose–response and time windows for effective treatment would also be useful. In a previous study assessing the effectiveness of a different collateral therapy, we found that delayed treatment after 90 min was not effective in SHRs (26). We therefore chose to do an early treatment in the current study in order to have an effect for comparison purposes. Finally, we used only male rats for this study because a previous study showed no differences in LMA function between sexes. However, sex differences in stroke outcomes are well-known and could impact the response to collateral therapy.

## Data availability statement

The original contributions presented in the study are included in the article/Supplementary material, further inquiries can be directed to the corresponding author.

## Ethics statement

The animal study was approved by University of Vermont Institutional Animal Care and Use Committee. The study was conducted in accordance with the local legislation and institutional requirements.

## Author contributions

MC: Conceptualization, Data curation, Formal analysis, Funding acquisition, Investigation, Methodology, Project administration, Resources, Supervision, Validation, Writing – original draft, Writing – review & editing. RH: Data curation, Formal analysis, Investigation, Methodology, Writing – review & editing. DL: Validation, Writing – review & editing. ST: Data curation, Formal analysis, Investigation, Methodology, Validation, Writing – review & editing.
