# Supplementary material for: The impact of collateral therapeutics on stroke hemodynamics in normotensive and hypertensive rats: a step toward translation
Source: Front Neurol. 2024 Mar 21;15:1373445. doi: 10.3389/fneur.2024.1373445 (PMC10996366; doi:10.3389/fneur.2024.1373445)
Supplement: Supplementary file 1 [file Data_Sheet_1.docx]

Supplementary Material

# Supplementary Figures


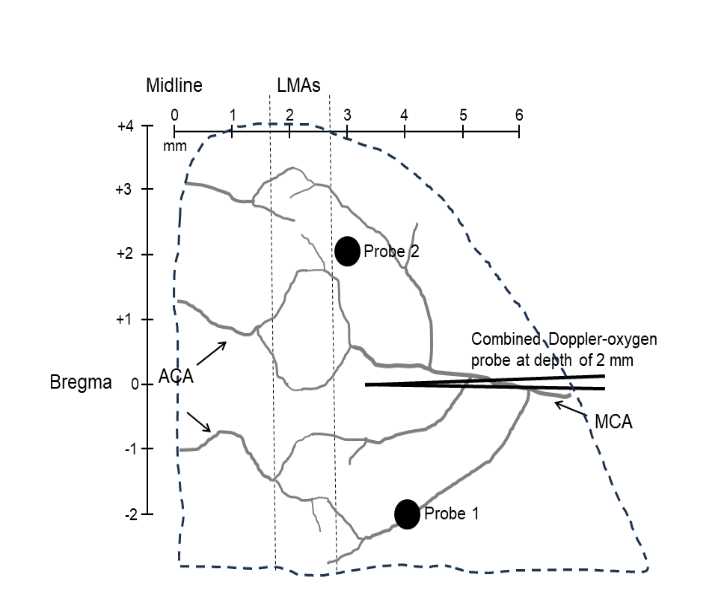

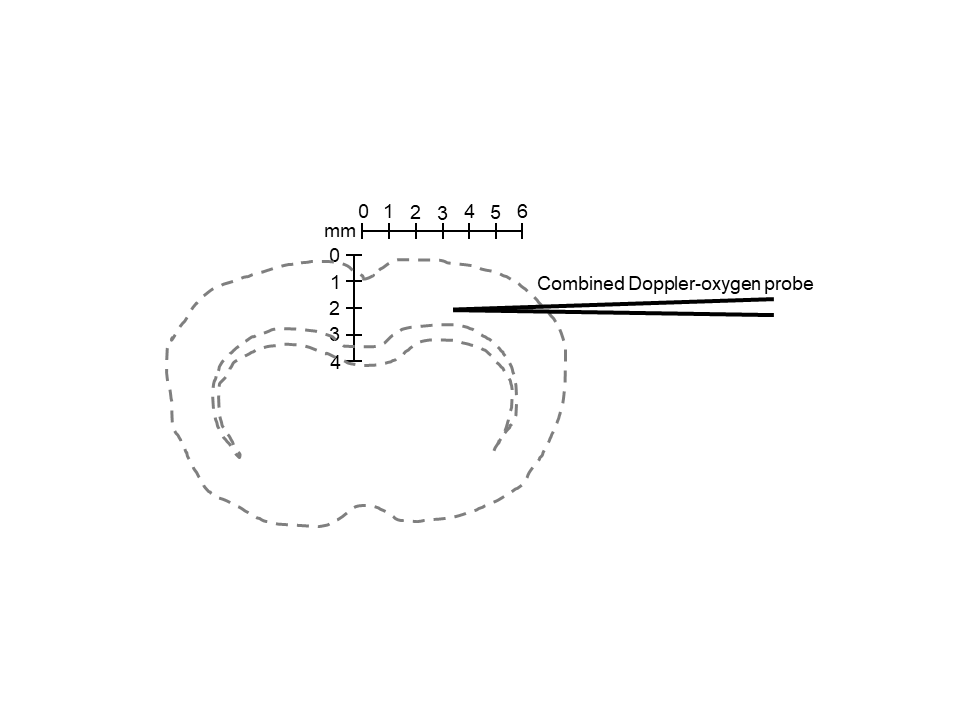


A.

B.

**Supplementary Figure 1**. Placement of probes for CBF and brain tissue oxygen measurements. (A) Skull coordinates for placement of surface Doppler probes used to measure changes in CBF in the core infarct territory (Probe 1) and through leptomeningeal anastomoses (LMAs) that connect distal branches of anterior (ACA) and middle (MCA) cerebral arteries (Probe 2). (Adapted from Chan et el. Stroke 2018; 49:1969-1976). Also shown is the placement of the combination Doppler-oxygen probe within the cortex. (B) Drawing showing coronal section of brain where the combination Doppler-oxygen probe was placed within the cortex. Scale shows skull coordinates.

**Supplementary Figure 2.** Raw CBF and blood pressure (BP) tracings for a) Wistar rats and b) SHR prior to, during and after pressor therapy by phenylephrine (PE) infusion to raise blood pressure. Top tracing shows middle cerebral artery (MCA) core perfusion (probe 1, see manuscript details for skull coordinates). Middle tracing shows collateral perfusion (probe 2, see manuscript details for skull coordinates). Bottom tracing shows blood pressure.


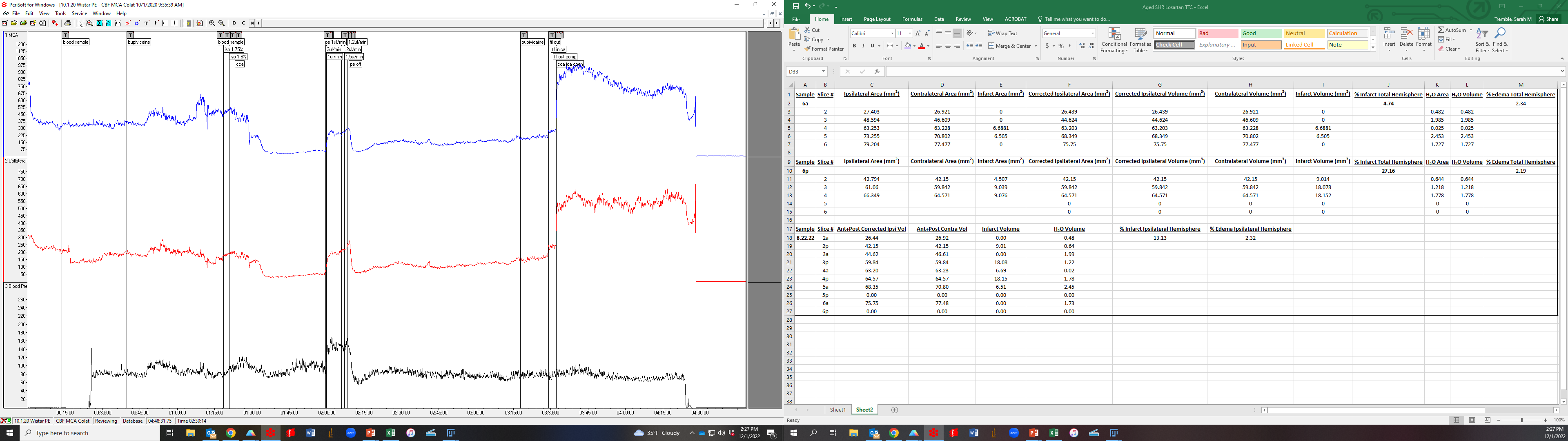


**Wistar – Pressor**

**a**


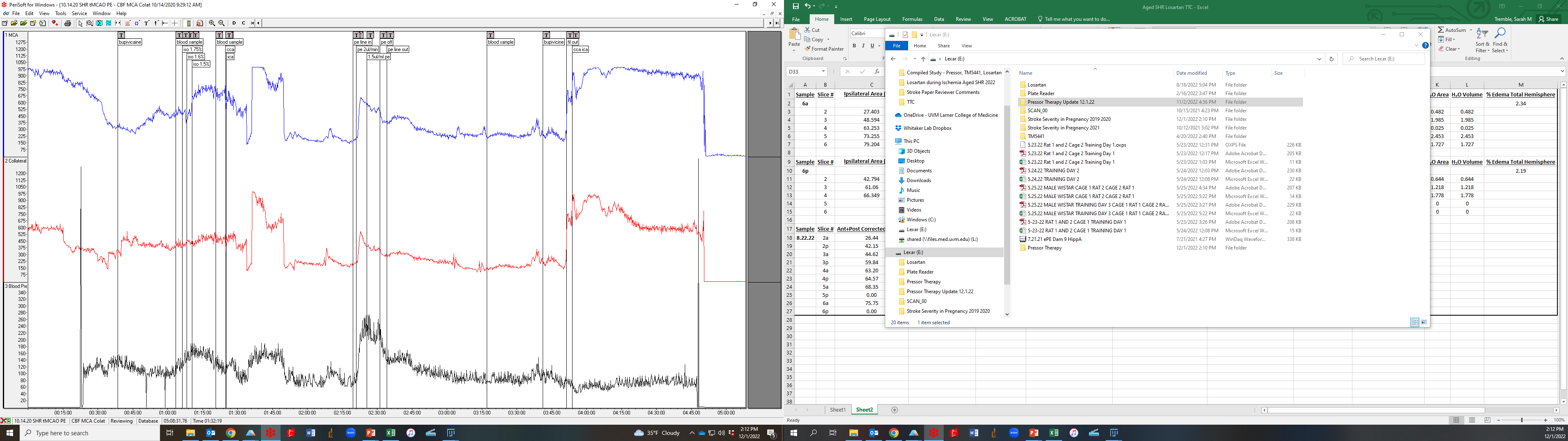


**SHR - Pressor**

**b**

**MCA CBF**

**(core)**

**MCA/ACA CBF**

**(collateral)**

**BP**

**PE**

**Reperf**

**PE**

**Reperf**

**Supplementary Figure 3**. Representative coronal sections stained with TTC for infarct and edema measurements from Wistar (left) and SHR (right) after pressor therapy.


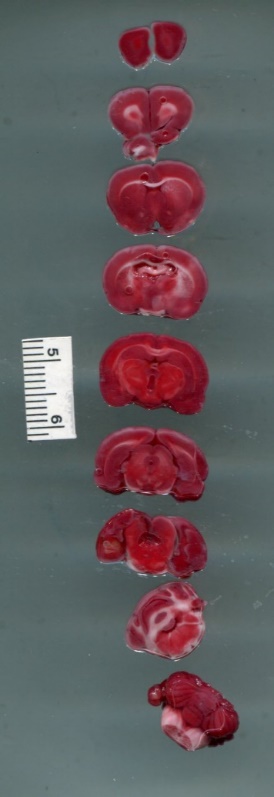


**Wistar + Pressor**


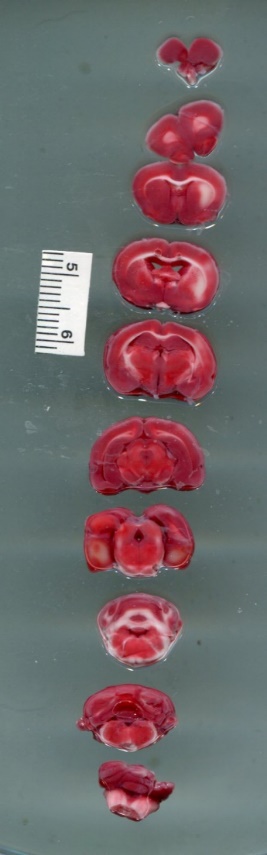


**SHR+ Pressor**

**Supplementary Figure 4.** Raw CBF and blood pressure (BP) tracings for a) Wistar rats and b) SHR prior to, during and after TM5441 infusion. Top tracing shows middle cerebral artery (MCA) core perfusion (probe 1, see manuscript details for skull coordinates). Middle tracing shows collateral perfusion (probe 2, see manuscript details for skull coordinates). Bottom tracing shows blood pressure.


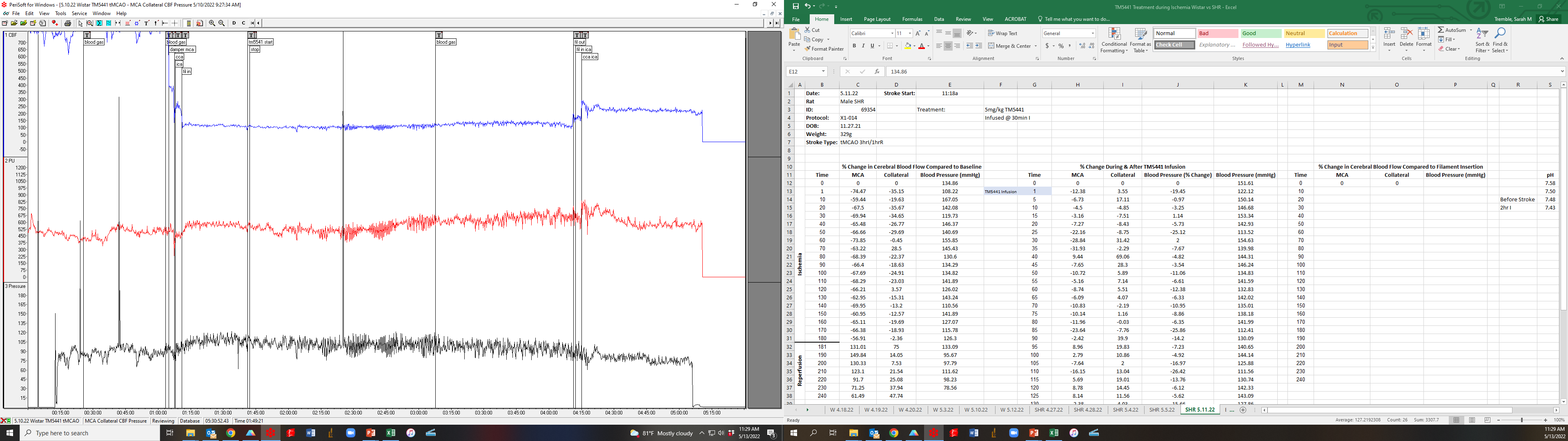


**Wistar - TM5441**


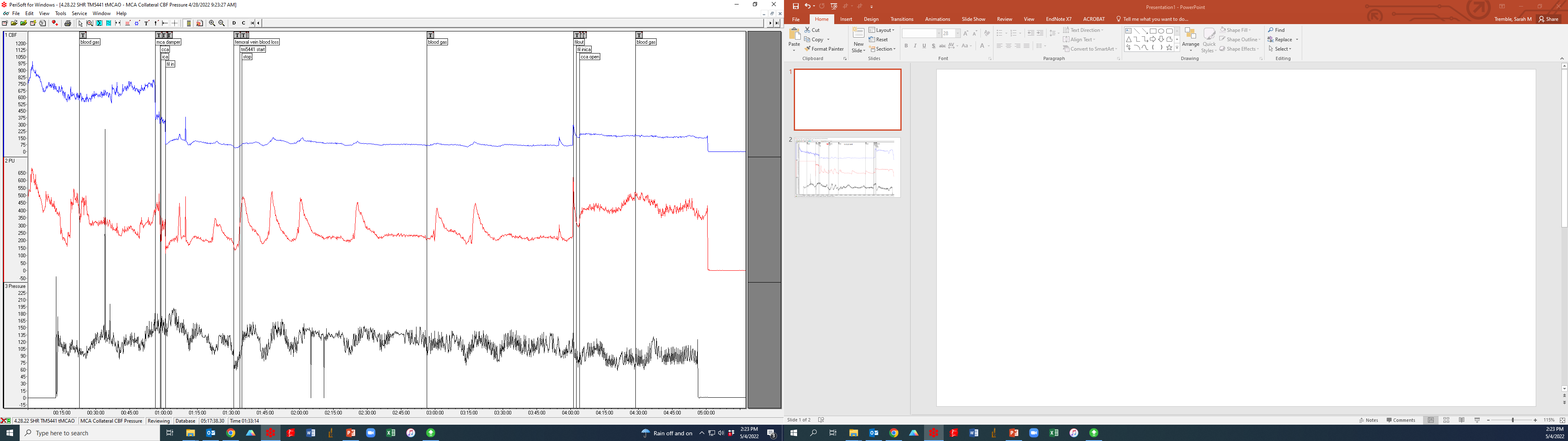


**SHR - TM5441**

**a**

**b**

**TM 5441**

**Reperf**

**MCA CBF**

**(core)**

**MCA/ACA CBF**

**(collateral)**

**BP**

**TM 5441**

**Reperf**

**Supplementary Figure 5**. Representative coronal sections stained with TTC for infarct and edema measurements from Wistar (left) and SHR (right) after TM5441 therapy.


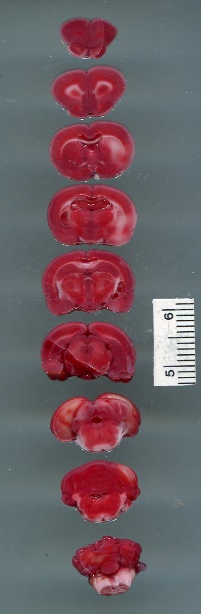


**Wistar +TM5441**


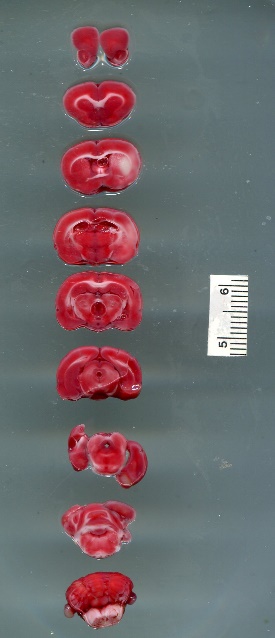


**SHR + TM5441**
